# The effect of fibroblast growth factor 21 on a mouse model of bovine viral diarrhea

**DOI:** 10.3389/fvets.2023.1104779

**Published:** 2023-03-09

**Authors:** Dan Zhao, Yu-Hao Song, Jin-Ming Song, Kun Shi, Jian-Ming Li, Nai-Chao Diao, Ying Zong, Fan-Li Zeng, Rui Du

**Affiliations:** ^1^College of Animal Science and Technology, Jilin Agricultural University, Changchun, China; ^2^College of Chinese Medicinal Materials, Jilin Agricultural University, Changchun, China; ^3^Jilin Province Sika Deer Efficient Breeding and Product Development Technology Engineering Research Center, Jilin Agricultural University, Changchun, China; ^4^The Ministry of Education Key Laboratory of Animal Production and the Product Quality and Safety, Jilin Agricultural University, Changchun, China

**Keywords:** bovine viral diarrhea, fibroblast growth factor 21, bovine viral diarrheal virus, duodenal epithelial cells, mouse model

## Abstract

Previously, we researched that bovine viral diarrhea virus (BVDV) induced a very significant increase in fibroblast growth factor 21 (FGF21) expression in mouse liver and that FGF21 was increased in the peripheral blood of BVD cattle and BVD mice. To determine the role of FGF21 in relieving clinical symptoms and inhibiting the intestinal damage caused by BVDV in BVD development in mice, BALB/c mice were intraperitoneally injected with cytopathic biotype (cp) BVDV-LS01 (isolated and identified by our group) to establish a BVD mouse model. The role of FGF21 in the BVD mouse model was investigated by injecting the mice with FGF21. The animals were divided into control, BVDV challenge, BVDV + FGF21, BVDV + FGF21Ab (anti-FGF21 antibody), and BVDV + IgG (immunoglobulin G) groups. The stool consistency, the degree of bloody diarrhea, histopathological changes, inflammatory cell infiltration, weight loss percentage, and detection of BVDV in the feces of the mice were examined, and the pathological changes and inflammatory cytokine expression were analyzed. The results showed that after BVDV challenge, the average BVD mouse model score of the BVDV mice was 11.6 points. In addition to mild diarrhea and tissue damage, BVDV was detected in the stools of 13 BVDV mice. Only two mice in the control group had scores (both, 1 point each). The comprehensive scoring results demonstrated the successful establishment of the BVD mouse model. FGF21 alleviated the clinical symptoms in the BVD mice and significantly improved weight loss. Furthermore, FGF21 inhibited the BVDV-induced leukocyte, platelet, and lymphocyte reduction while inhibiting the expression of BVDV-induced inflammatory factors. In the BVD mice, FGF21 promoted duodenal epithelial cell proliferation, thereby significantly improving the damage to the cells. In conclusion, FGF21 exerted a good therapeutic effect on the BVD mouse model.

## 1. Introduction

Bovine viral diarrhea (BVD) is an acute, highly contagious disease caused by BVD virus (BVDV). BVD is widely prevalent worldwide and causes huge economic losses to the global livestock industry. The main clinical symptoms of BVD are subclinical infection, reproductive failure, respiratory and intestinal diseases due to immunosuppression, thrombocytopenia, and bleeding, and fatal mucosal disease, which is one of the most severe clinical forms of BVDV infection, with mortality rates as high as 100% (1). In addition to mainly infecting cattle, pigs, sheep, goats, and deer, camels and other wild animals are also susceptible hosts for BVDV, which causes serious harm to them (2, 3). Along with border disease virus and classical swine fever virus, BVDV is a single-stranded RNA virus that belongs to the family *Flaviviridae*, genus *Pestivirus*. BVDV genotypes are classified into BVDV1, BVDV2, and HoBi-like viruses based on differences in their 5′ untranslated region, Npro, or E2 genes (HoBi-like viruses are listed as BVDV3, but the International Committee on Taxonomy of Viruses has not confirmed the BVDV3 viral typing) (4). Each genotype is further divided into multiple sub-genotypes. At least 21 BVDV1 sub-genotypes (1a−1u), four BVDV2 sub-genotypes (2a−2d), and four HoBi-like sub-genotypes have been identified (3, 5, 6). The main prevalent BVDV types in China are BVDV2 and BVDV1, of which the main subtypes are BVDV1a and BVDV1b (7). BVDV is divided into the cytopathic biotype (cp) and the non-cp (ncp) based on whether it can cause cellular pathological changes (8–10).

Fibroblast growth factor 21 (FGF21) is a secreted protein with multiple biological functions. Nobuyuki Itoh's team successfully cloned the *FGF21* gene in 2000 (11). In 2005, Kharitonenkov et al., scientists at the Lilly laboratory in the United States, reported the role of FGF21 in diabetes treatment and described the biological significance of FGF21 protein as a new metabolic regulator (12). FGF21 contains 210 amino acids, is highly conserved in mammals, and has a molecular weight of −22.3 kDa. Human FGF21 contains 209 amino acids and its coding sequence is very similar to that of mice (75% homology) (13). FGF21 is mainly expressed in liver tissue; it does not bind to heparan sulfate protein glycans or has a very low affinity for them, which enables its circulatory transport and endocrine action. Research on FGF21 in animal and human metabolic diseases such as obesity and diabetes are relatively mature (14) and FGF21 is closely related to diseases or pathological processes such as metabolic syndrome and non-alcoholic fatty liver (15, 16). Recent studies reported that FGF21 expression is significantly increased in diverse inflammatory diseases (17–19). These findings suggested that, as a biomarker, FGF21 is becoming a research hotspot for targeted drugs for various metabolic diseases. Many current studies reported that FGF21 is significantly elevated in the peripheral blood of patients with hepatitis B, hepatitis C, and HIV (20). FGF21 is closely correlated with disease severity, suggesting that it has an antiviral effect, but its mechanism has not been thoroughly explored.

Previously, we researched that BVDV induced a very significant increase in FGF21 expression in mouse liver and that it played an endocrine role in blood circulation. Furthermore, FGF21 expression was increased in the peripheral blood of BVD cattle and BVD mice. Currently, few studies have reported on FGF21 in viral diseases. Therefore, to determine the role of FGF21 in relieving the clinical symptoms and inhibiting the intestinal damage caused by BVDV in BVD development in mice, we established a BVD mouse model by infecting BALB/c mice with the BVDV-LS01 strain we previously isolated and identified, and injected the BVD mice with recombinant FGF21 protein. The mechanism of FGF21 in the BVD mice was identified based on changes in the clinical symptoms, body weight, histopathology, complete blood count (CBC), and cellular inflammatory factors. This study provided evidence for new research directions regarding the role of FGF21 in viral diseases and its application in BVD adjuvant therapy and prevention and control.

## 2. Materials and methods

### 2.1. Experimental design and BVD mouse model establishment

This study was conducted after receiving Jilin Agriculture University Institutional Animal Care and Use Committee approval (JLAU08201409). The experimental procedures were performed in compliance with the National Institutes of Health Guide for the Care and Use of Laboratory Animals (NIH Publications No. 8,023).

The model objects were 6–8-week-old male BALB/c mice [animal license number: SCXK (Jing) 2019–0008, purchased from Beijing Huafukang Biotechnology Co., Ltd., Beijing, China] (21). In total, 75 mice were used (15 mice per group). The mice were randomly divided into five groups. Group A mice were injected with Dulbecco's modified Eagle's medium (DMEM) as the negative control group. Group B–E mice were injected intraperitoneally with 0.3 mL tissue culture fluid [median tissue culture infective dose (TCID_50_) = 10^6.6^/0.1 mL containing BVDV-LS01]. Group B (BVDV challenge, BVDV) comprised BVD mice. Group C (BVDV + FGF21) mice were injected intraperitoneally with mouse recombinant FGF21 protein [injection dose, 4 mg/kg/day (22)] donated by the Jilin Institute of Agricultural Science and Technology on day 6 of BVDV challenge. From day 4 of the experiment onwards, group D (BVDV + FGF21Ab) mice were intraperitoneally injected with 5 mg/kg anti-FGF21 antibody once every other day, while group E (BVDV + IgG) mice were intraperitoneally injected with 5 mg/kg immunoglobulin G (IgG) antibody every other day (23).

The clinical status of the mice was observed every day after the challenge. The consistency of the mouse feces, presence of bloody stool, tissue damage, inflammatory cell infiltration, and fecal virus detection were recorded. The observations were scored according to the disease scoring standard, where a comprehensive score ≥ 8 points (including BVDV detection) indicated that the mouse qualified as a BVD mouse model. Tables 1, 2 depict the groupings and disease scoring criteria, respectively. The mice were culled when they exhibited obvious clinical symptoms, and the role of FGF21 in the mice was subsequently analyzed from the clinical symptom, tissue change, and inflammatory factor aspects.

**Table 1:** BVD mouse model construction.

**Group**	**Number of mice**	**Immunogen**
A	15	DMEM
B	15	LS01
C	15	LS01 + FGF21
D	15	LS01 + FGF21Ab
E	15	LS01 + IgG

**Table 2:** Disease activity scoring criteria.

**Score per line item (points)**	**Stool consistency**	**Blood in stool**	**Tissue damage**	**Inflammatory cell infiltration**	**Weight loss (%)**	**BVDV in stool and tissue**
0	Normal	None	Intestinal mucosa not damaged	Small number of inflammatory cells in the lamina propria	0	No
1	Slightly soft	–	Intestinal epithelial cell damage	Mucosal lamina propria invaded by a large number of inflammatory cells	<3	–
2	Very soft	Occult blood	Intestinal mucosal damage or focal ulceration	Extensive inflammatory cells and extension into the submucosa	3–5	–
3	Loose	–	Intestinal mucosal damage and spread to deeper intestinal wall structures	Massive inflammatory cell invasion of the submucosa	5–10	–
4	–	Visible blood in stool	–	–	>10	Yes

The overall score is composed of the total score of the sum of the individual scores; “–” indicates non-scoring items.

### 2.2. Clinical symptoms and body weight changes in BVD mouse model

The diet, metabolism, and mental state of the mice were observed daily after BVDV challenge, and the mouse body weight changes were recorded. Whether FGF21 played a role in BVD development was evaluated based on the clinical symptoms and body weight changes.

### 2.3. BVDV detection in BVD mouse tissues and organs

The mice were observed daily for clinical symptoms and were killed when they exhibited listlessness, rough coat, decreased diet, shapeless feces, or obvious diarrhea. Subsequently, blood was collected from the eyes, and the kidney, spleen, liver, duodenum, and colon tissues were collected.

The fresh feces (500 mg) was obtained from the mouse rectum and soaked in 200 μL pre-cooled diethylpyrocarbonate (DEPC) water for 15 min to dissolve the virus contained in the feces. The DEPC water was centrifuged at 4°C at 10,000 × *g* for 5 min and the supernatant was stored for subsequent use.

The tissue samples (100 mg) was ground into powder with liquid nitrogen, and 1 mL pre-cooled saline was added. After repeated freeze-thawing, the sample was centrifuged at 10,000 × *g* at 4°C for 5 min, and the supernatant was obtained.

Total RNA was extracted from the fecal and tissues samples with an RNA extraction kit (Takara Bio, Shiga, Japan). Complementary DNA (cDNA) was synthesized by reverse transcription and the BVDV content was analyzed by qRT-PCR using a Prime Direct™ Probe RT-qPCR Mix (Takara Bio). The relative BVDV content was calculated in relation to that of the normal control. Table 3 shows the primers used in this study.

**Table 3:** Primer sequences used for PCR.

**Primer**	**Sequence (5^′^ → 3^′^)**
BVDV-F	CCATGCCCTTAGTAGGACTAG
BVDV-R	CTCCATGTGCCATGTACAGCAG
IL6-F	ATGAAGTTCCTCTCTGCAAGAGAC
IL6-R	CACTAGGTTTGCCGAGTAGATCTC
IL1β-F	TTCATCTTTGAAGAAGAGCCCAT
IL1β-R	TCGGAGCCTGTAGTGCAGTT
MCP1-F	GGCTCAGCCAGATGCAGT
MCP1-R	GAGCTTGGTGACAAAAACTACAG
TNFα-F	CACCACCATCAAGGACTCAA
TNFα-R	AGGCAACCTGACCACTCTCC

### 2.4. Histopathological examination of the duodenum

The BVD mouse model exhibited obvious duodenal lesions and relatively severe tissue damage. Therefore, we performed an in-depth study focusing on the duodenal lesions after FGF21 injection. Part of the duodenum was fixed in 4% formaldehyde, paraffin-embedded, and sectioned to 6-μm thickness. The tissue sections were stained with hematoxylin–eosin (H&E) and for E-cadherin for pathological analysis.

### 2.5. CBC testing

The peripheral blood was collected from the inferior vena cava and anticoagulant (trisodium citrate) was added to the collection tube. The platelets, leukocytes, and lymphocytes were analyzed with a CBC analytical instrument.

### 2.6. Analysis of inflammatory cytokines in the blood

Peripheral blood that had been collected in non-anticoagulant-containing blood collection tubes was centrifuged at 1,500 × *g* for 30 min at 4°C to obtain the plasma. The cytokines IL-6, TNF-α, and MCP1 were measured from the cell-free supernatants using enzyme-linked immunosorbent assay kits (Becton, Dickinson and Company, America).

### 2.7. *In vitro* culture of duodenal tissue and analysis of inflammatory cytokines

The intestinal contents were cleaned from 2-cm long mouse duodenum samples, the tissue opened longitudinally, soaked in phosphate-buffered saline (PBS, containing streptomycin and penicillin) and washed for 1–2 min, then washed 5–6 times in antibiotic-free RPMI 1640 medium. Subsequently, the tissue was cut into 1-cm^2^ pieces, then incubated in antibiotic-free RPMI 1640 medium at 37°C for 24 h.

Total RNA from virus-infected duodenum cells was extracted using an RNA extraction kit following the instructions for specific operations. cDNA was synthesized by reverse transcription according to the PrimeScript™ RT Reagent Kit. The reverse transcription condition was 42°C for 1.5 h while the PCR conditions were 94°C for 30 s (one cycle), followed by 95°C for 15 s and 58°C for 1 min for 40 cycles. Blank control wells were set up for each amplification and each experiment was repeated no less than three times. The internal reference gene was β-actin. The target genes were quantified using the relative quantitative analysis method (comparative threshold cycle [2^−Δ*ΔCt*^] method). Table 3 lists the sequences of the primers used.

### 2.8. Analysis of FGF21 promotion of duodenal epithelial cell proliferation

To verify whether FGF21 was involved in duodenal epithelial cell proliferation and regeneration, the BVDV-infected mice were injected intraperitoneally with 30 mg/kg 5-bromodeoxyuridine (BrdU) at 2 h, 24 h, and 48 h before the end of BVDV infection.

### 2.9. Statistical analysis

The data of each group are expressed as the mean ± SEM. Statistical analysis was performed with one-way analysis of variance using GraphPad Prism 8.0.2 (GraphPad Software Inc., La Jolla, CA, USA). The mean between two groups was compared using the *t*-test. *P* < 0.05, *p* < 0.01, and *p* < 0.001 indicated a statistically significant difference, highly statistically significant difference, and statistically extremely significant difference, respectively.

## Results

### 3.1. FGF21 injection alleviated clinical symptoms in mice after BVDV challenge

**Figure 1:**
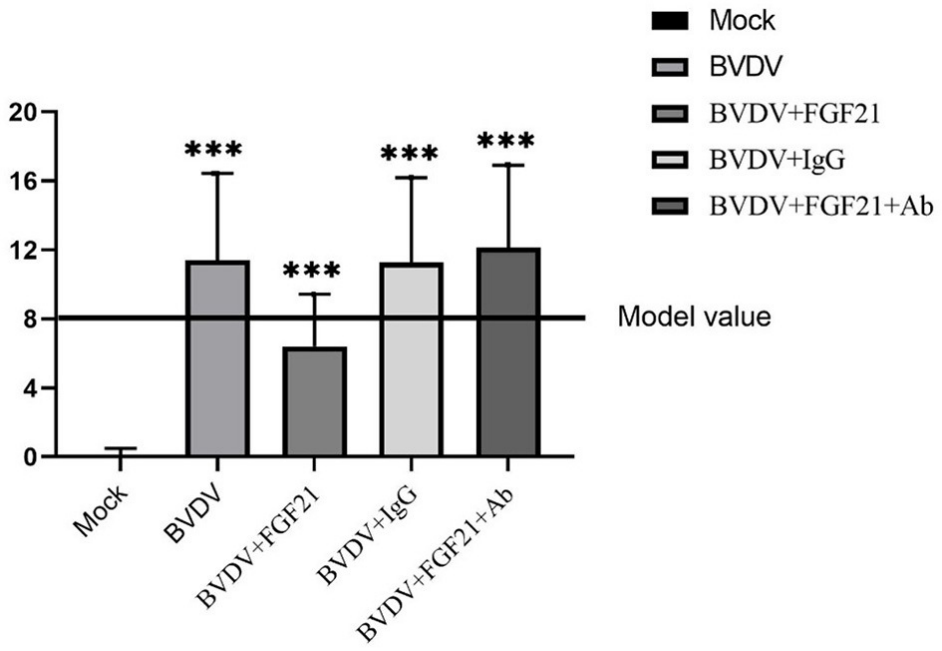
BVD mouse model score statistics.

Score statistics were performed according to the BVD mouse model scoring criteria. Figure 1 depicts the results. The mean statistical scores of the BVDV and BVDV + IgG groups were 11.4 and 11.3, respectively. On day 3 after challenge, some mice exhibited signs of lethargy, rough coats, mild diarrhea, eating less, and crowding. The autopsy results of these mice demonstrated a few hemorrhagic spots of different sizes in the liver and lung and obviously enlarged spleen, mesenteric lymph nodes, and paraduodenal Peyer patches.

The BVDV + FGF21Ab mice exhibited more obvious clinical symptoms, where the average statistical score was 12.1 points. Some mice developed obvious diarrhea and lethargy on day 2 after FGF21Ab injection. The autopsy results of the BVDV + FGF21Ab mice revealed a few hemorrhagic spots in the liver and lung and obviously swollen spleen, mesenteric lymph nodes, and paraduodenal Peyer patches.

The BVDV + FGF21 group had an average statistical score of 6.4 points. The BVDV + FGF21 mice had basically normal food intake, smooth fur, no diarrhea symptoms, no hemorrhagic spots in the liver and lungs, and slight swelling of the spleen.

The average statistical score of the control mice was 0.13 points, and they exhibited no obvious clinical symptoms or pathological changes throughout experiment.

### 3.2. FGF21 injection slowed BVDV challenge-induced weight loss

**Figure 2:**
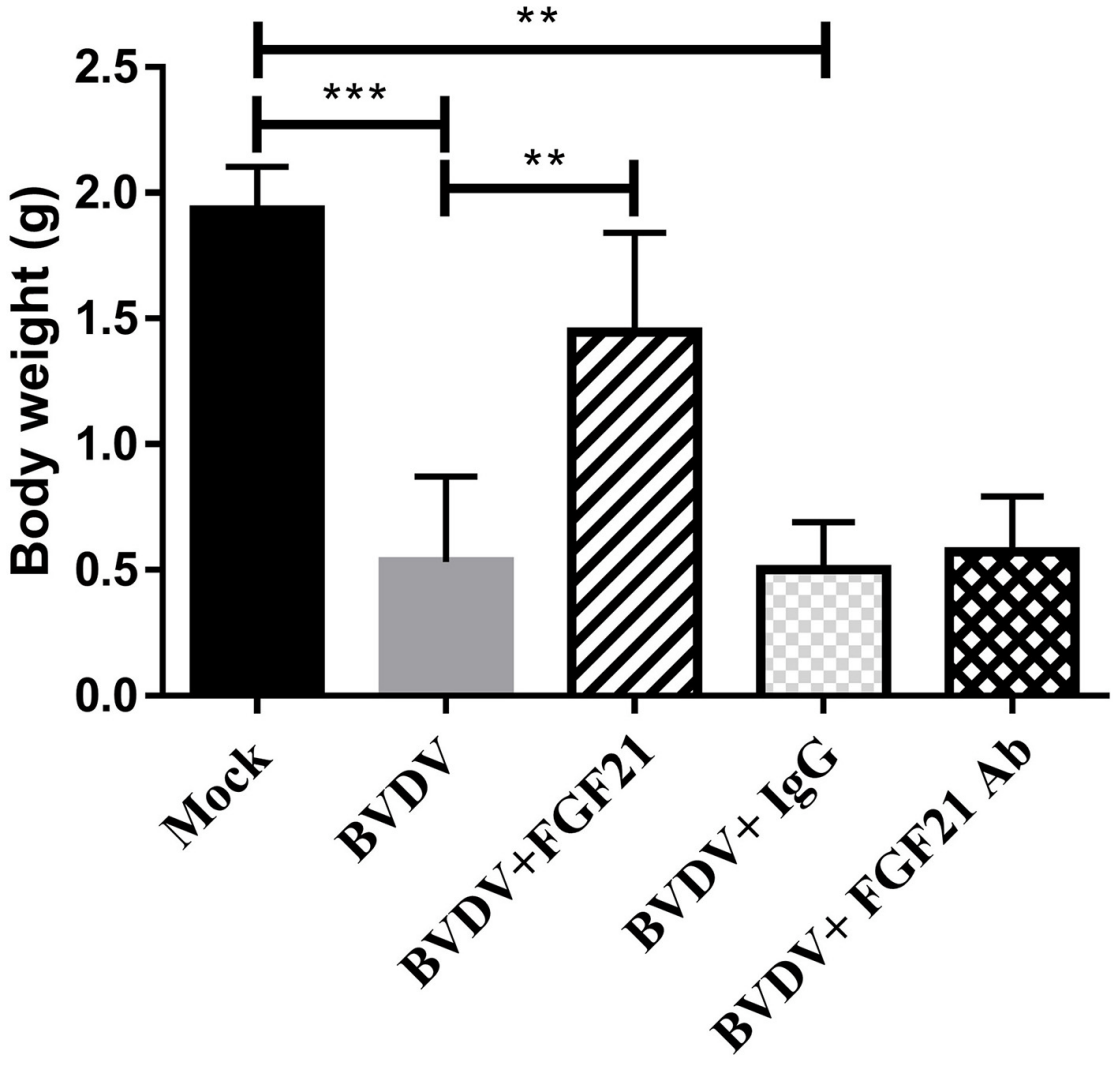
Mouse body weight changes.

The mice were weighed and recorded daily; Figure 2 depicts weight changes. Throughout the experiment, there were extremely significant differences in the body weight changes between the control and BVDV groups and the BVDV + IgG and BVDV + FGF21Ab groups (*p* < 0.001). The BVDV + FGF21 group recorded significantly different weight gain compared the BVDV and BVDV + IgG groups (*p* < 0.01). The results indicated that FGF21 attenuated weight loss in the BVD mice during BVD development.

### 3.3. BVDV detection results in mice after challenge

**Figure 3:**
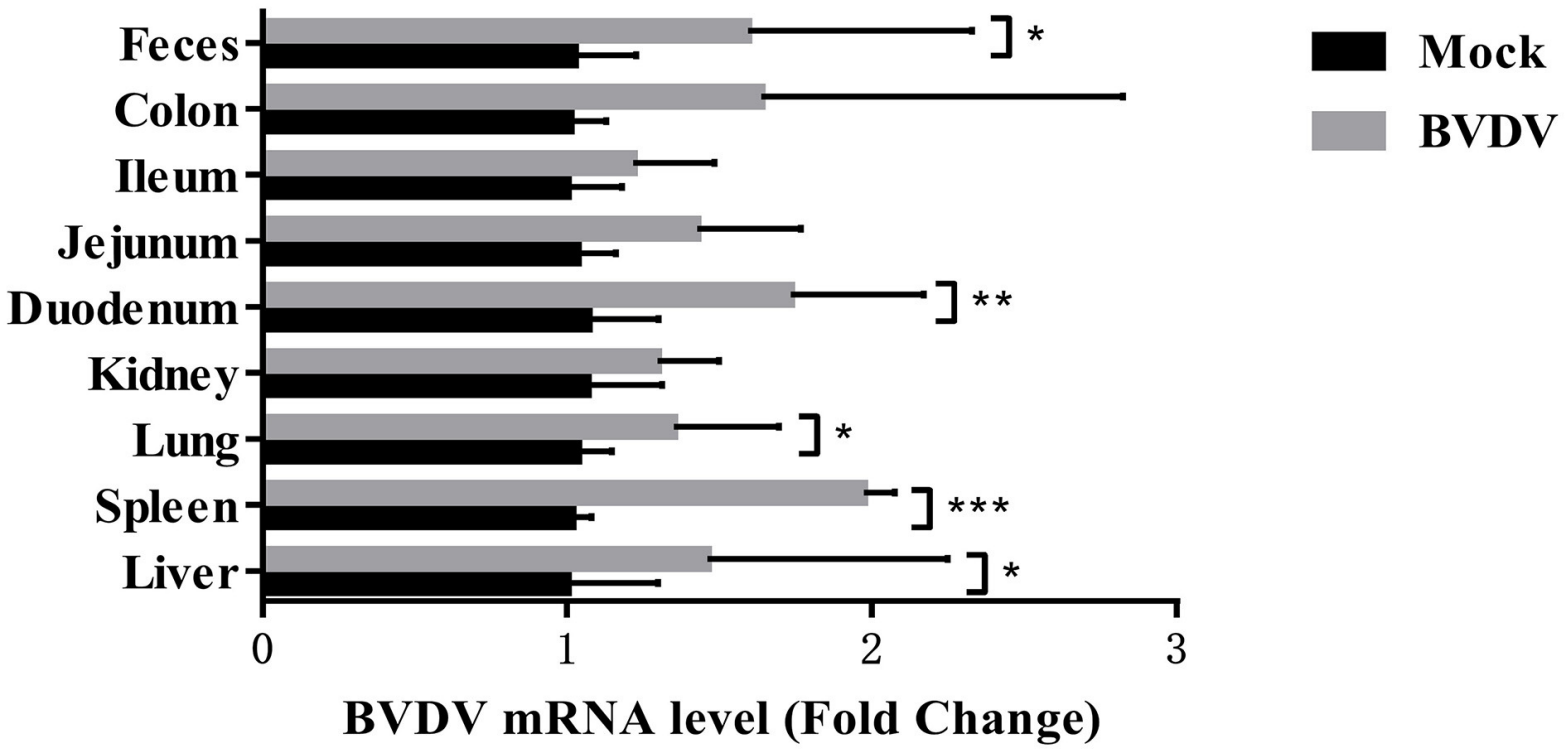
BVDV levels in mouse tissues.

Figure 3 depicts the BVDV detection results in the mice after challenge. Ten days after BVDV challenge, the BVDV mice exhibited obvious clinical symptoms such as loose stool and diarrhea. BVDV was detected in the mouse liver, lung, spleen, kidney, duodenum, colon, jejunum, ileum, and feces. The BVDV content was highest in the mouse spleen, followed by that in the duodenum, colon, and feces.

### 3.4. CBC analysis

**Figure 4:**
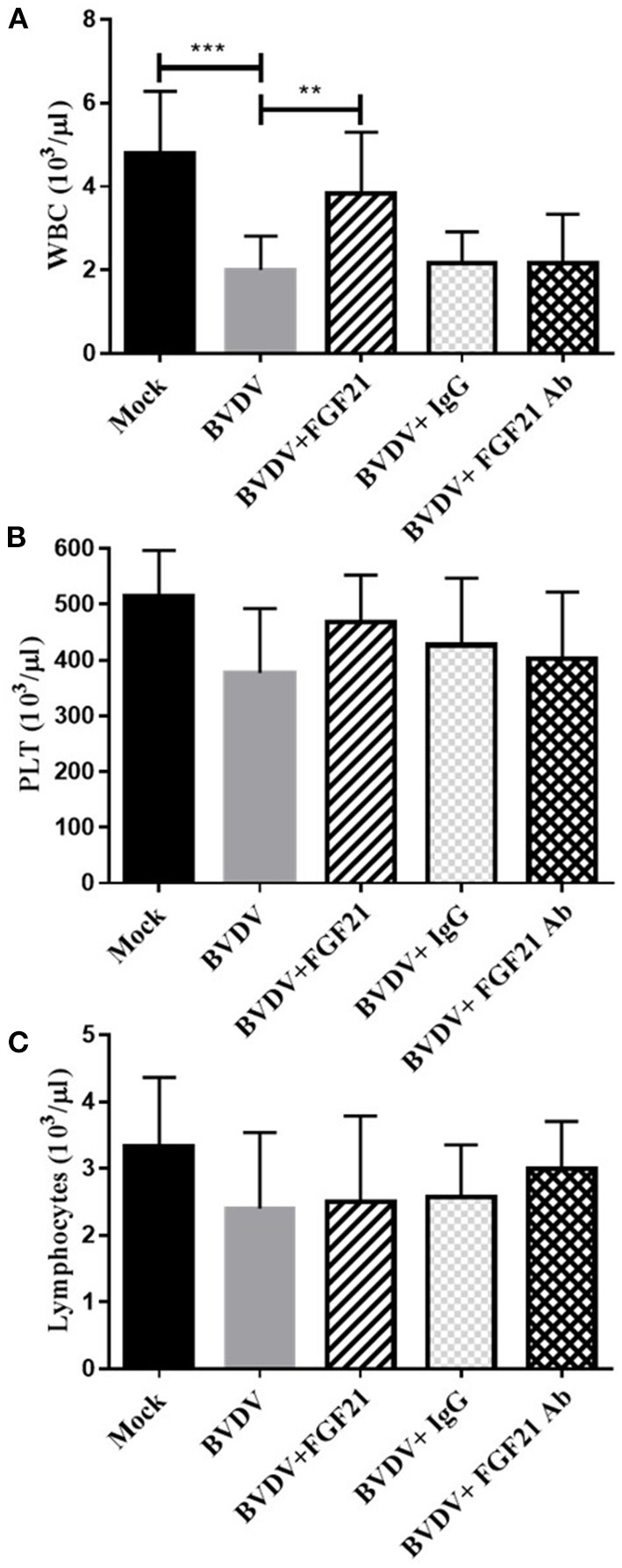
Alterations in mouse leukocyte (WBC) **(A)**, platelet (PLT) **(B)**, and lymphocyte **(C)** levels.

Figure 4 depicts the leukocyte, platelet, and lymphocyte levels in the mouse blood after challenge. The BVDV, BVDV + IgG, and BVDV + FGF21Ab mice had significantly decreased leukocyte, platelet, and lymphocyte levels (*p* < 0.001). The platelet and lymphocyte contents in the BVDV, BVDV + FGF21Ab, and BVDV + IgG groups were not significantly changed, while the leukocyte contents increased significantly after FGF21 injection (*p* < 0.01).

### 3.5. Pathological changes of the duodenum

**Figure 5:**
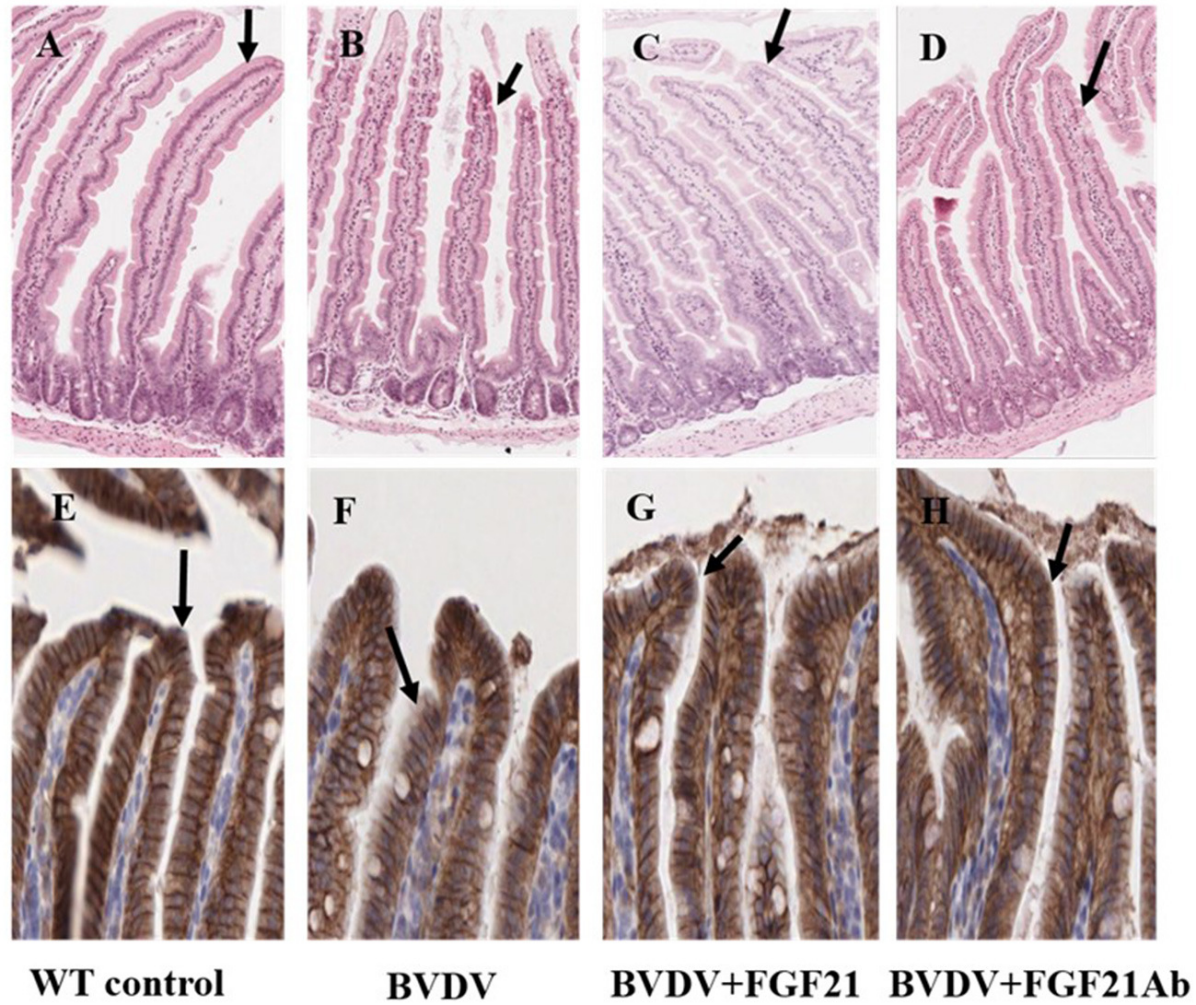
Main pathological changes in the mice (H&E staining, × 100; E-cadherin staining, × 200). **(A–D)** H&E staining of duodenal epithelial cells in the control **(A)**, BVDV **(B)**, FGF21 **(C)**, and BVDV+FGF21Ab groups **(D)**. **(E–H)** E-cadherin staining of duodenum in the control **(E)**, BVDV **(F)**, FGF21 **(G)**, and BVDV+FGF21Ab groups **(H)**.

Figure 5 depicts the histopathological observations of the H&E-stained mouse duodenal tissue. The control group demonstrated intact duodenal epithelial phenotype, good crypt structure, and no lymphocyte infiltration in the mucosa. The duodenal tissue of the BVDV and BVDV + FGF21Ab mice exhibited edema, local epithelial ulcers, submucosal inflammatory cell infiltration, and duodenal villus epithelium shedding after challenge. However, injection of the FGF21 recombinant protein was followed by subsiding duodenal intestinal wall edema, significantly relieved ulcer, and the epithelium was significantly improved and tended to be complete.

The changes in the mouse duodenal injury were observed using E-cadherin staining, which demonstrated that E-cadherin expression was reduced. The duodenal villous epithelium of the BVDV and BVDV + FGF21Ab mice was damaged and shed after challenge, and the intestinal epithelium was seriously damaged after FGF21Ab injection. After FGF21 recombinant protein injection, the intestinal tract was obviously improved and tended to be complete.

### 3.6. FGF21 inhibited the expression of BVDV-induced inflammatory factors

**Figure 6:**
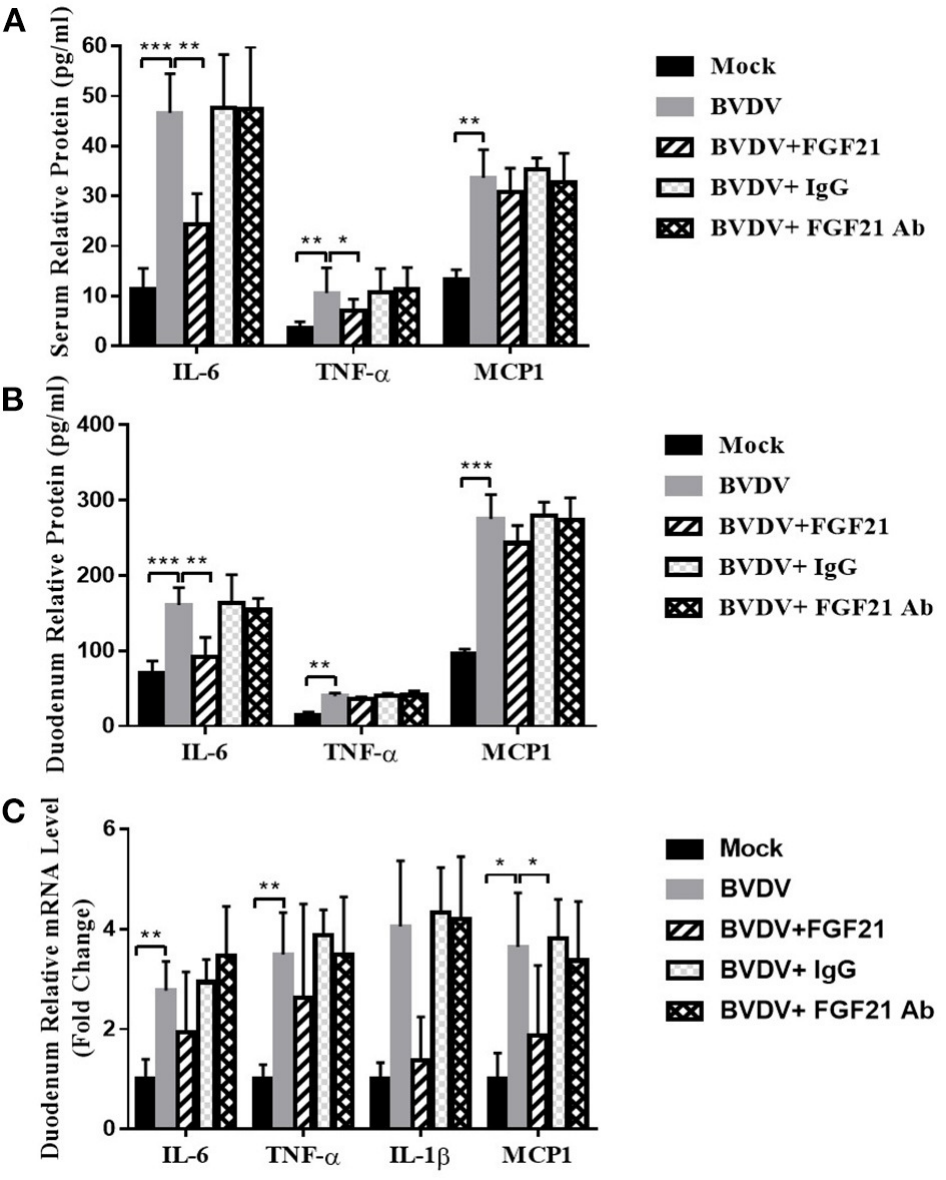
Mouse plasma and duodenum levels of inflammatory cytokines. **(A, B)** Inflammatory cytokine levels in the plasma **(A)** and the duodenum **(B)**. **(C)** Inflammatory cytokine mRNA levels in the duodenum.

Figure 6 depicts the results of FGF21 inhibition of BVDV-induced inflammatory factor expression. After challenge, the peripheral blood of the BVDV, BVDV + FGF21Ab, and BVDV + IgG mice contained significantly increased TNF-α, MCP1 (both, *p* < 0.01), and IL-6 levels (*p* < 0.001). However, after FGF21 injection, the peripheral blood IL-6 and TNF-α levels were significantly decreased (*p* < 0.01 and *p* < 0.05, respectively), while MCP1 levels were decreased but not significantly (Figure 6A).

After the duodenal tissue had been cultured *in vitro* for 24 h, the proinflammatory cytokine content in the culture supernatant was detected by ELISA. The results demonstrated that the BVDV, BVDV + FGF21Ab, and BVDV + IgG mice produced significantly more IL-6, TNF-α, and MCP1 than the control group (*p* < 0.001). However, after the FGF21 injection, the BVDV + FGF21 mice had significantly decreased duodenal IL-6 content (*p* < 0.01) while the TNF-α and MCP1 contents were not significantly decreased (Figure 6B).

Similar to the results of the peripheral blood and duodenal tissue *in vitro* culture, the duodenal IL-6, TNF-α, and IL-1β mRNA levels in the BVD, BVDV + FGF21Ab, and BVDV + IgG mice were significantly increased (*p* < 0.01), as was MCP1 (*p* < 0.05). After the FGF21 injection, the IL-6, TNF-α, IL-1β, and MCP1 mRNA levels were all decreased in the BVDV + FGF21 group, but only the MCP1 decrease was significantly different (*p* < 0.05) (Figure 6C).

### 3.7. FGF21 promoted duodenal epithelial cell proliferation

**Figure 7:**
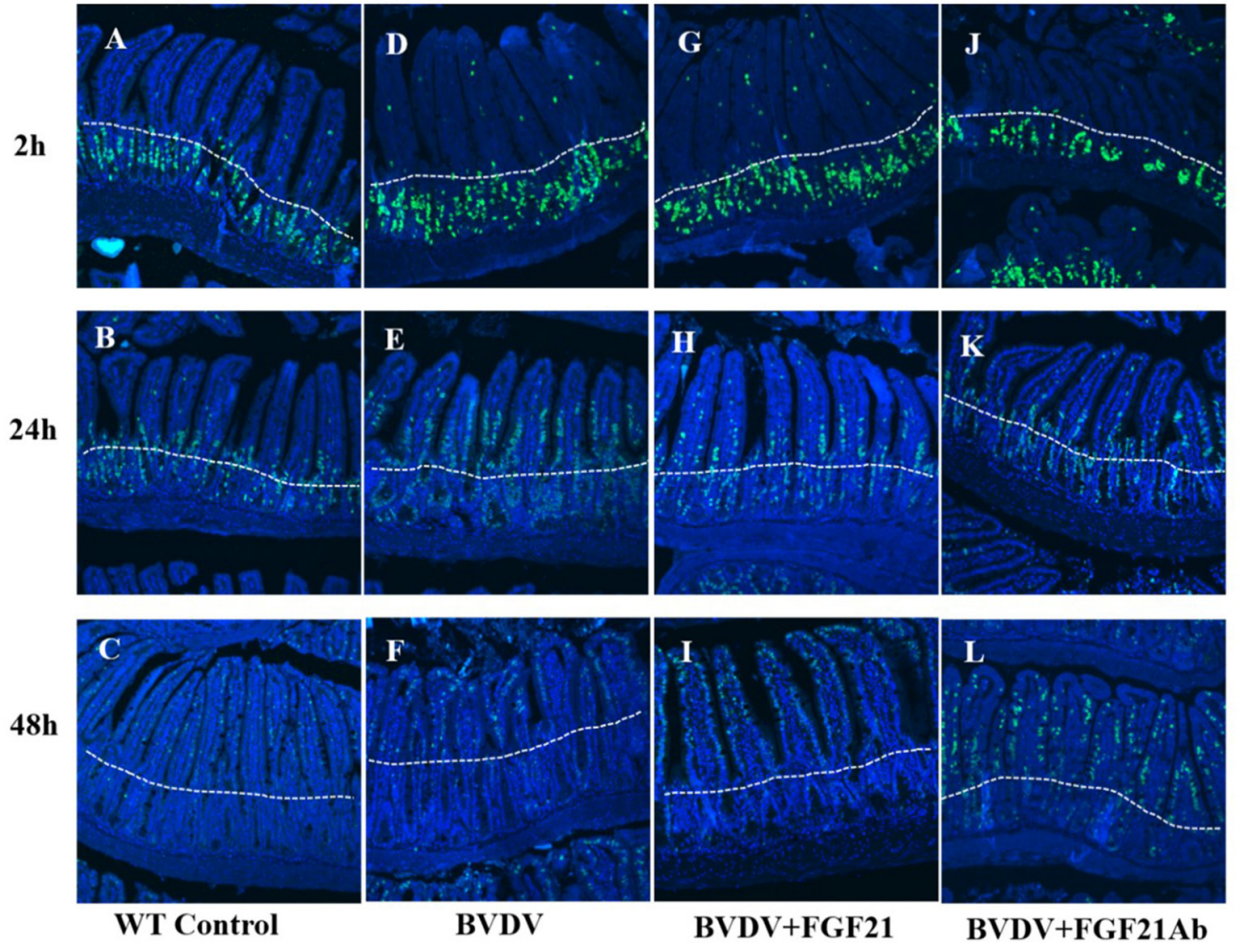
BrdU staining of mouse duodenal epithelial cells. **(A–C)** BrdU staining results at 2 h **(A)**, 24 h **(B)**, and 48 h **(C)** in the control group. **(D–F)** BrdU staining results at 2 h **(D)**, 24 h **(E)**, and 48 h **(F)** in the BVDV group. **(G–I)** BrdU staining results at 2 h **(G)**, 24 h **(H)**, and 48 h **(I)** in the FGF21 group. **(J–L)** BrdU staining results at 2 h **(J)**, 24 h **(K)**, and 48 h **(L)** in the BVDV+FGF21Ab group.

Figure 7 depicts the results of FGF21 promotion of duodenal epithelial cell proliferation. BVD mice injected with FGF21 recombinant protein demonstrated a significantly larger number of BrdU-positive duodenal intestinal epithelial cells than the control mice.

## 4. Discussion

Previously, we determined that FGF21 expression in BVD bovine and BVD mouse peripheral blood exhibited the same upward trend, indicating that BVDV infection in animals stimulates high FGF21 expression. These results were similar to that of Xia et al. regarding FGF21 and ulcerative colitis mice (24). To determine how FGF21 functions in BVD mice, we performed animal experiments based on a BVD mouse model, where BVD mice were injected intraperitoneally with mouse FGF21Ab, IgG, or recombinant FGF21 protein. The results demonstrated that after BVDV challenge, the BVDV and BVDV + IgG groups essentially exhibited the same clinical symptoms and necropsy changes. The results revealed FGF21 delayed or alleviated the clinical symptoms in the BVD mice and significantly improved weight loss, which provided a characterization basis for the role of FGF21.

Diarrhea is one of the main clinical symptoms of BVD. The main pathological changes are hemorrhage spots of different sizes in the intestinal mucosa; swollen and bleeding mesenteric lymph nodes (25–29); intestinal wall thickening; intestinal lymph node enlargement; necrosis and shedding of mucosal epithelial cells in the small intestine, cecum, and colon; lymphocytic infiltration of the mucosal lamina propria; hyaline degeneration and fibrinoid necrosis of the submucosal arteries; crypt epithelial shedding; and vacuolization (28, 29). The histopathological observation revealed that the BVD mice had hemorrhage spots in the intestinal mucosa and lymphocyte infiltration in the mucosal lamina propria, especially duodenal epithelial cell necrosis and shedding, which was consistent with the previous study (30). Gumbiner (31) reported that E-cadherin is a calcium-dependent cell adhesion protein expressed in epithelial cells that is critical for maintaining intestinal epithelial integrity and is indirectly involved in defense against enteropathogens. Stephane et al. (32) reported that the loss of E-cadherin expression led to the loss of adherens junctions and desmosomes, thereby resulting in apoptosis and cell shedding. E-cadherin function in the gut has been implicated in pathological processes. The E-cadherin staining results in this study demonstrated that E-cadherin expression was decreased in the duodenal epithelial cells of the BVDV and BVDV + FGF21Ab mice. FGF21 injection promoted duodenal epithelial cell proliferation and significantly improved the damage to the BVD mouse duodenal epithelial cells.

Proinflammatory cytokines such as IL-6, TNF-α, and MCP1 are key in the pathogenesis of intestinal inflammation. Diarrhea caused by intestinal inflammation is one of the main clinical symptoms of BVD. The autopsy results of the BVD mice demonstrated that duodenal inflammation was the most serious symptom. Therefore, the cytokine levels in the peripheral blood and duodenal tissue of the mice in all groups were detected, where IL-6, TNF-α, and MCP1 in the peripheral blood and duodenum of the BVD mice were significantly increased (*p* < 0.01). Specifically, IL-6 was extremely significantly increased (*p* < 0.001). The IL-6 and TNF-α mRNA levels were highly significantly increased in the duodenum of BVD mice (*p* < 0.01). Furthermore, the leukocyte, platelet, and lymphocyte numbers in the peripheral blood of the BVD mice were decreased, and the leukopenia was highly significantly different (*p* < 0.01), which indicated that BVDV caused intestinal-related inflammation in the mice, which resulted in epithelial cell damage.

However, after FGF21 injection, the peripheral blood leukocytes of the BVD mice were significantly increased to normal levels. Notably, IL-6 in the peripheral blood and duodenum was highly significantly decreased (*p* < 0.01). While TNF-α and MCP1 were not significantly different, *MCP1* mRNA levels in the duodenum were significantly different (*p* < 0.05). The results proved that FGF21 inhibited the BVDV-induced inflammatory factor expression. This result was similar to that of Singhal et al. (33) and Johnson et al. (34), who reported that FGF21 might be important in inhibiting and regulating inflammation, respectively.

Our CBC test results were consistent with those of a previous study that reported that different BVDV strains can cause leukocyte, lymphocyte, and platelet reduction in mouse peripheral blood (35). Generally, virus infections cause leukocyte reduction in the peripheral blood. In our experiment, the leukocyte levels returned to the normal range after FGF21 injection.

Although we only focused on the related inflammatory factors in the duodenum with obvious tissue lesions and analysis of the clinical symptoms, body weight changes, CBC testing, histopathology, and changes of inflammatory factors in the BVD mice, the results proved that FGF21 is involved in the improvement and treatment of BVD occurrence and development in mice. The results provided new ideas for BVD prevention and treatment and further research.

## 5. Conclusions

We successfully established a BVD mouse model. FGF21 injection alleviated the clinical symptoms of BVD in the mice and significantly improved the weight loss in the BVD mice. FGF21 inhibited BVDV and reduced leukocyte, platelet, and lymphocyte levels in the peripheral blood. Furthermore, FGF21 inhibited the expression of BVDV-induced inflammatory factors. Moreover, FGF21 promoted duodenal epithelial cell proliferation and significantly improved the damage to the duodenal epithelial cells in the BVD mice. In conclusion, FGF21 exerted a good therapeutic effect on the BVD mouse model.

## Data availability statement

The original contributions presented in the study are included in the article/supplementary material, further inquiries can be directed to the corresponding authors.

## Ethics statement

This study was conducted following recommendations of the Jilin Agriculture University Institutional Animal Care and Use Committee (JLAU08201409) and the experimental procedures were performed in compliance with the National Institutes of Health Guide for the Care and Use of Laboratory Animals (NIH Publications No. 8023).

## Author contributions

DZ, Y-HS, and J-MS designed the study. KS, J-ML, N-CD, and YZ assisted with data analysis. DZ, F-LZ, and RD performed animal tests, interpreted the results, and wrote the manuscript. All authors have read and approved the final version of manuscript.
